# Exploration of Whitlockite Nanostructures for Hemostatic Applications

**DOI:** 10.7759/cureus.58701

**Published:** 2024-04-21

**Authors:** Abhay Kumar Jain V, Saheb Ali, Ramadurai Murugan, Chitra S

**Affiliations:** 1 Pharmacology, Saveetha Medical College and Hospital, Saveetha Institute of Medical and Technical Sciences, Saveetha University, Chennai, IND; 2 Periodontics, Saveetha Dental College and Hospitals, Saveetha Institute of Medical and Technical Sciences, Saveetha University, Chennai, IND; 3 Center for Global Health Research, Saveetha Medical College and Hospital, Saveetha Institute of Medical and Technical Sciences, Saveetha University, Chennai, IND; 4 Prosthodontics, Saveetha Dental College and Hospital, Saveetha Institute of Medical and Technical Sciences, Saveetha University, Chennai, IND

**Keywords:** health, regeneration, blood coagulation, hemostasis, whitlockite

## Abstract

Background

Calcium magnesium phosphate (CMP)-based whitlockite is a promising biomaterial for hemostasis and regenerative applications. Regenerative approaches aim to advance tissue repair and recovery in different clinical scenarios. Whitlockite is a biocompatible and biodegradable mineral that has garnered impressive consideration for its interesting properties, making it an appealing candidate for therapeutic applications.

Aim

This study aimed to evaluate the hemostatic behavior of synthesized whitlockite nanoparticles.

Materials and methods

Coprecipitation and hydrothermal methods were used to synthesize whitlockite nanoparticles. Calcium nitrate, magnesium nitrate, and diammonium hydrogen phosphate were used as precursors to prepare this material.

Results

Crystalline phases of whitlockite (Ca_3_Mg)_3_(PO_4_) and calcium magnesium phosphate Ca_7_Mg_2_P_6_O_2_ were observed through X-ray diffraction (XRD) patterns, along with relevant properties of the phosphate functional group detected through Raman spectra. This study explores the hemostatic adequacy of CMP-based whitlockite using different methodologies. The capacity of the materials to actuate platelet conglomeration and encourage clot arrangement is assessed using *in vitro *experiments. Moreover, this study investigates the regenerative potential of CMP-based whitlockite in tissue-building applications.

Conclusion

The structural and morphological parameters provide crucial insights into the proper formation of the material, and the hemoclot assessment aids in understanding its coagulation behavior. Future investigations and clinical trials will be instrumental in fully harnessing the potential of CMP-based whitlockite for advancing hemostasis and regenerative medicine.

## Introduction

Mineral apatite is a crystalline form of calcium phosphate and is a naturally occurring hydroxyapatite. It is the primary mineral in mammalian bones and teeth, giving those structures their strength and rigidity. Ca_5_(PO_4_)_3_(OH) is the chemical formula for hydroxyapatite, which is made up of atoms of calcium, phosphorus, oxygen, and hydrogen arranged in a particular crystal structure [1]. Due to its biocompatibility and capacity to integrate with natural tissues, hydroxyapatite is also utilized in several biomedical applications, such as tissue engineering scaffolds, drug delivery systems, and dental implants [2]. To be used in these applications, it can also be synthesized in a lab [3]. Hydroxyapatite is a renowned material for its capacity to support tissue regeneration and biocompatibility [4].

Hydroxyapatite is frequently used as a scaffold material in the field of tissue engineering to support the growth of new tissue [5]. Therefore, cells can adhere and multiply on the surface of the scaffold due to the structural resemblance between hydroxyapatite and natural bone [6]. New tissue can be created due to the cell's ability to add extracellular matrix to the scaffold gradually. Additionally, hydroxyapatite has been applied to dentistry [7]. Tricalcium phosphate and calcium magnesium phosphate are two essential minerals that are essential for bone and tissue regeneration [8]. The calcium that makes up a large portion of these structures is responsible for maintaining the strength and rigidity of the bones and teeth [9]. Calcium is essential for the development of new bone tissue when tissue regeneration is taking place. The proliferation and differentiation of osteoblasts and the cells responsible for bone formation are two cellular processes that depend on calcium ions and are crucial for bone growth and healing [10]. Another mineral that is critical for tissue regeneration is magnesium. It participates in various cellular functions, such as cell division, protein synthesis, and DNA synthesis [11]. Another mineral required for bone and tissue regeneration is phosphate. It contributes significantly to the mineral hydroxyapatite, which constitutes the majority of bone tissue.

Tricalcium phosphate can be resorbed over time as new bone tissue develops due to its biocompatibility, which means it does not trigger an immune reaction or exhibit toxicity within the body [12]. In conclusion, crucial minerals for bone and tissue regeneration include calcium, magnesium, phosphate, and tricalcium phosphate. They can be used in various regenerative medicine applications to support the growth of new bone by playing critical roles in cellular processes vital for bone growth and healing [13]. Due to its distinct characteristics, whitlockite (WH), a rare calcium magnesium phosphate mineral, has recently attracted interest in the field of biomedical research. WH has the potential to be used in a variety of biomedical processes, such as cancer treatment, drug delivery, and bone tissue engineering [14]. WH biocompatibility prevents an immune reaction or toxicity in the body, which is one of its key characteristics. It is, therefore, a material that shows promise for use in biomedical applications [15]. WH is a desirable substance for bone tissue engineering applications because of its similar composition to the mineral phase found in natural bone.

The current approaches for the development of WH for biomedical applications include its synthesis using different techniques, modification of its surface properties to enhance its interaction with biological molecules and cells, and investigation of its potential use as a biomaterial for bone tissue engineering and regeneration as well as hemostatic applications [16]. Selecting a synthesis methodology is critical when designing WH for biomedical applications. It is essential to optimize the synthesis parameters to achieve the desired properties, such as particle size, crystal structure, purity, and surface properties, for the intended biomedical application.

## Materials and methods

**Figure 1:**
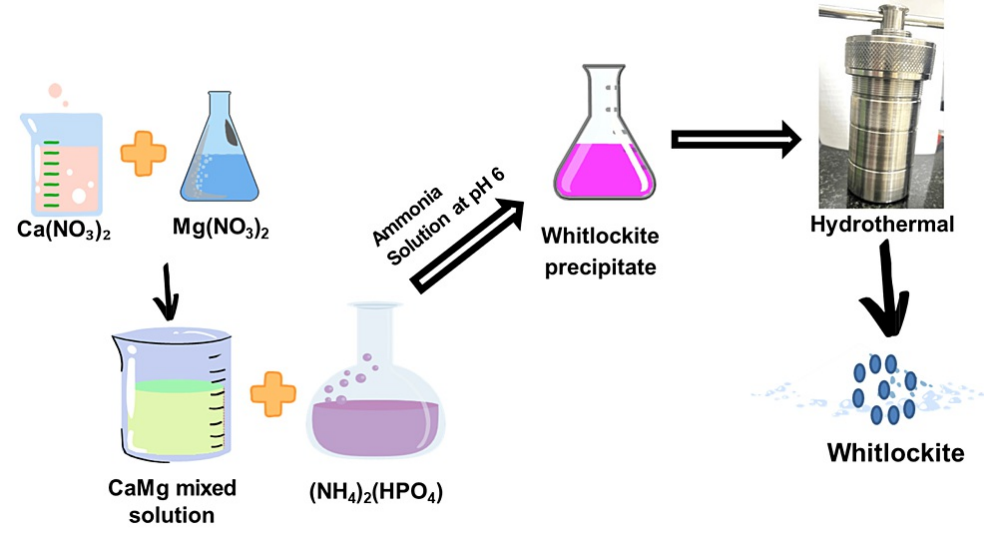
Synthesis of whitlockite. The scheme represents the synthesis of whitlockite using the hydrothermal method. Without the hydrothermal setup, the dried powder is obtained through the coprecipitation method. Figure credits: Chitra Shivalingam.

The precipitation method was employed to synthesize the WH nanoparticles. Calcium nitrate (0.5 M/25 mL) was mixed with magnesium nitrate (0.5 M/3.5 mL) to form a calcium magnesium solution. After mixing the solution, diammonium hydrogen phosphate (0.5 M/20 mL) was added dropwise, and the pH of the solution was maintained at 6 using an ammonia solution. Then, the precipitated particles were collected and dried using a hot air oven (100 °C for 12 hours), along with the co-precipitated particles. For hydrothermally derived particles, the precipitates were placed in a hydrothermal setup and kept in a hot air oven for 15 minutes at a temperature of 200 °C to obtain WH powder. After cooling down the temperature, the obtained particles were washed with deionized water, followed by ethanol, and then dried in a hot air oven at 100 °C overnight to collect the nanoparticles, as depicted in Figure 1.

Characterization techniques

To investigate the characteristics of the materials and evaluate the crystalline phases, X-ray diffraction (XRD) was used with the Cu Kα wavelength (Bruker D8 advance, Billerica, Massachusetts). Raman spectroscopy (WITEC ALPHA300 RA-Confocal Raman-AFM Microscope, Ulm, Germany) was used to examine the characteristics of functional groups. JEOL (JSM-IT 800, Tokyo, Japan) and Oxford Instruments (Abingdon, England) were utilized to analyze the morphological and elemental analysis.

## Results

XRD patterns

**Figure 2:**
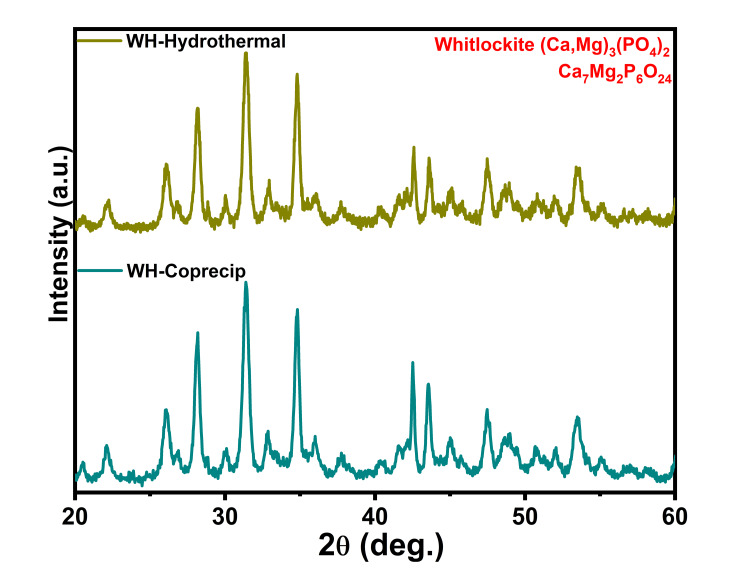
XRD patterns of whitlockite. XRD patterns of whitlockite by the coprecipitation and hydrothermal methods. XRD, X-ray diffraction.

The XRD diffractogram of WH depicted the well-matched crystalline phases of WH (Ca,Mg)_3_(PO_4_)_2_ (ICDD:00-013-0404) and calcium magnesium phosphate Ca_7_Mg_2_P_6_O_24_ (ICDD:00-020-0348) (Figure 2). Tricalcium phosphate (TCP) and magnesium-substituted tricalcium phosphate (TCMP) possess very close crystalline properties to WH. Magnesium is deposited in the cite of calcium in the TCP structure; however, in the case of WH, Mg^2+^ can also be incorporated in HPO_4_^2- ^[17]. Most literature reported Ca(OH)_2_ and Mg(OH)_2_ crystalline phases for WH. However, this study found calcium magnesium phosphate crystalline phases [18]. Similarly, Ca_18_Mg_2_(HPO_4_)_2_(PO_4_)_12 _is the predominant crystalline phase for WH, with a polygonal structure [19]. Current results showed the dominance of (Ca,Mg)_3_(PO_4_) with nanoparticles.

Raman spectra

**Figure 3:**
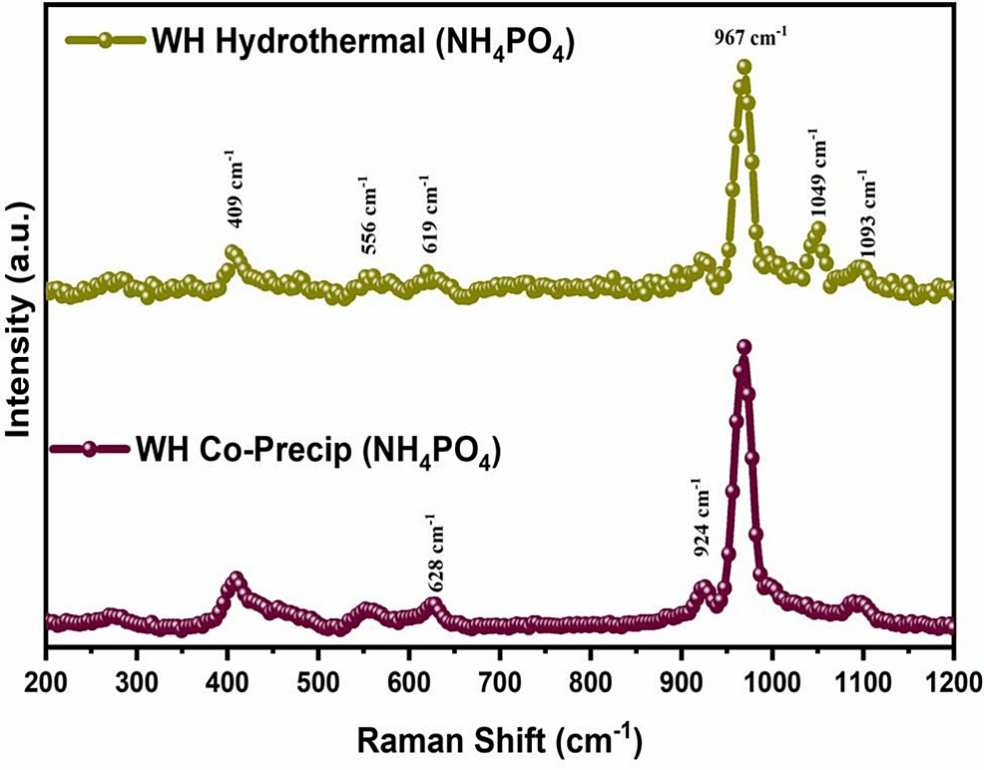
Raman spectra of whitlockite. Raman spectra of whitlockite by the coprecipitation and hydrothermal methods.

The Raman spectroscopic analysis of the WH showed specific peaks at 967 and 409 cm^−1^ (Figure 3), which are the characteristic peaks of υ1 and υ4 PO_4_^3−^ ions, while the peak at 409 cm^−1^ is a characteristic peak of υ2 PO_4_^3−^. This authenticates the prominent presence of phosphate and further reconfirms the presence of WH. The characteristic peaks of bone mineral apatite were observed at 959-975 cm^-1^ with sharp vibrations. The obtained peak broadening indicates the formation of WH [20]. The shoulder peak before 967 cm^-1^ indicates the proper formation of WH [21]. Hence, the obtained results indicate the formation of WH.

Field emission scanning electron microscopy (FE-SEM) and elemental analysis

**Figure 4:**
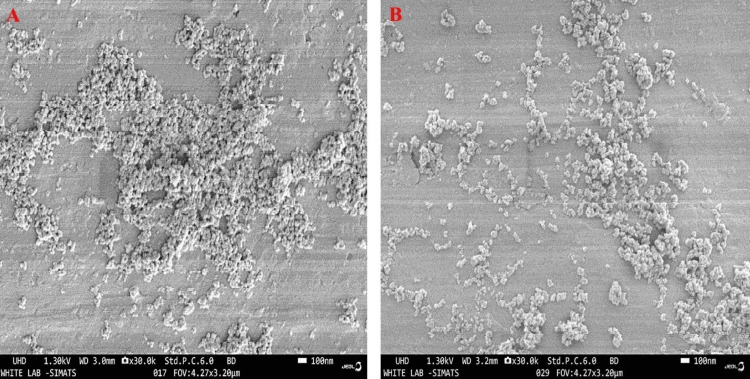
Morphology of whitlockite by field emission scanning electron microscopy. Morphology of whitlockite using field emission scanning electron microscopy by coprecipitation and hydrothermal methods. (A) WH - Hydrothermal and (B) WH - Coprecip.

The WH nanoparticles synthesized using the hydrothermal method showed an elongated, spherical shape; similarly, the WH obtained from coprecipitation showed a small spherical morphology (Figure 4). Uniform temperature and pressure are crucial for promoting the homogeneous growth of elongated, spherical structures in the hydrothermal method. Generally, WH tends to grow in a distinctive polygonal shape [22]; however, in this case, a spherical morphology was distinctly formed. Rod-like, hexagonal, and polygonal shapes are the most commonly observed structures for WH [17,19]; however, in this case, a spherical morphology was predominant. The Zn-infused WH showed highly agglomerated hexagonal particles measuring approximately 80 nm in size. The current results indicated that the particles are nearly homogeneous in size, measuring approximately 40 nm [23].

**Figure 5:**
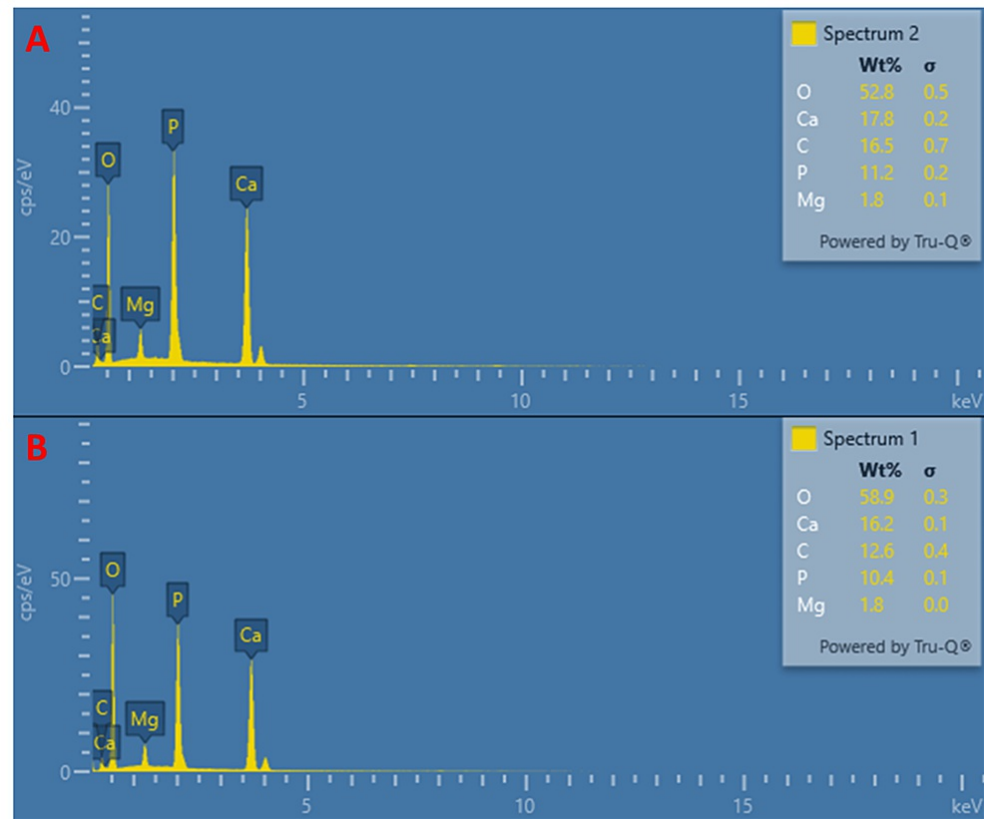
The elemental composition of the prepared material was confirmed by EDS and elemental mapping. The elemental composition of the prepared material (by coprecipitation and hydrothermal methods) was confirmed by energy dispersive spectroscopy (EDS) and elemental mapping: (A) hydrothermal and (B) coprecipitation.

**Figure 6:**
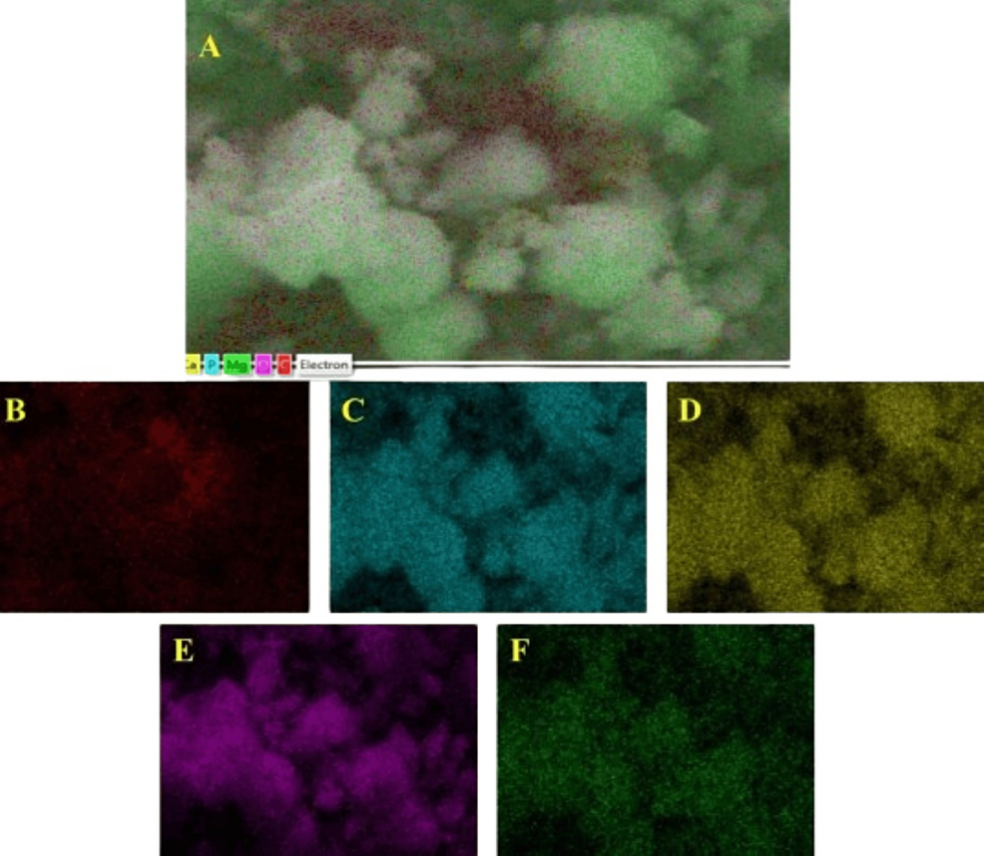
Elemental mapping of the synthesized particles using the hydrothermal method. The elemental mapping data of whitlockite synthesized using the hydrothermal method: (A) a mixture of all the elements, (B) carbon, (C) phosphate, (D) calcium, (E) oxygen, and (F) magnesium.

**Figure 7:**
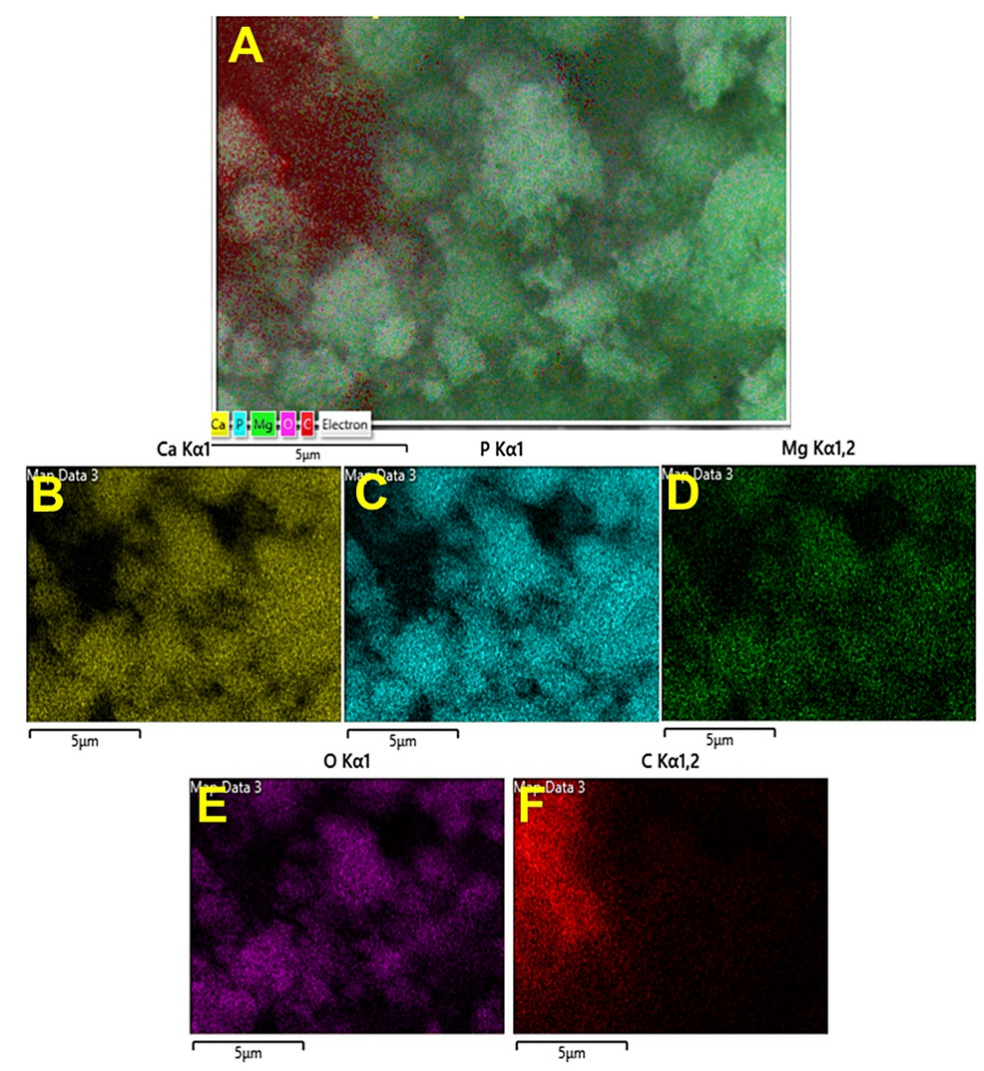
Elemental mapping of the synthesized particles using the coprecipitation method. The elemental mapping data of whitlockite synthesized using the coprecipitation method: (A) a mixture of all the elements, (B) calcium, (C) phosphate, (D) magnesium, (E) oxygen, and (F) carbon.

The elemental composition was confirmed through energy dispersive spectroscopy (EDS) and mapping spectra, revealing the presence of O, Ca, C, P, and Mg elements in both materials (Figures 5-7). Ca and P were consistently distributed throughout; Mg was also evenly dispersed on the nanoparticles. Based on these results, it can be concluded that nanoparticles contain the WH phase.

Hemostatic assay through FE-SEM

**Figure 8:**
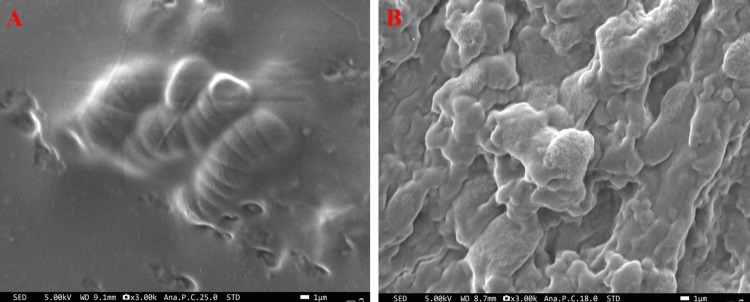
Hemostatic property of whitlockite. The hemostatic behavior of the blood was evaluated under two conditions: (A) control and (B) treatment with hydrothermally synthesized whitlockite.

The normal hemostasis has no control over death during significant medical procedures and injuries. Subsequently, external hemostatic agents are utilized to help typical coagulation pathways and to control bleeding. These external specialists are monetarily accessible in different forms. The most ordinarily utilized hemostatic specialists are comprised of chitosan [24]. The hemostatic behavior of the blood was evaluated with the treatment of hydrothermally synthesized WH compared to control; rapid clotting behavior was observed in treated blood samples (Figure 6). The osteo-conductive and osteo-inductive properties of calcium phosphate have been widely used in bone regeneration applications [25]. In order to promote bone regeneration, calcium and phosphorus ions are released, which controls how osteoblasts and osteoclasts are activated, as shown in Figure 8 [26]. The control of calcium phosphate surface characteristics and porosity affects cell/protein adhesion and growth and manages the production of bone minerals [27]. Due to the differences in ion release, solubility, stability, and mechanical strength, different types of calcium phosphate, including hydroxyapatite and TCP, have different properties affecting their bioactivity that can be used in various biomedical applications [28].

## Discussion

CMP-based WH altogether advances cell expansion, separation, and extracellular framework blend, demonstrating its capacity to support tissue regeneration. In addition, the biocompatibility and biodegradability of CMP-based WH are broadly examined to guarantee its safety and long-term use in the human body [29]. Cytotoxicity studies, histological assessments, and contamination examinations demonstrate that CMP-based WH shows favorable tissue regeneration [30]. In conclusion, this study presents CMP-based WH as a flexible biomaterial with dual benefits: promoting successful hemostatic properties and advancing tissue recovery [16]. It is also a promising candidate for various restorative applications, including wound administration, surgical strategies, and tissue regeneration procedures. CMP-based WH is highly effective for hemostasis and regenerative potential. They have versatile applications in regenerative medicine applications, aiding in the growth of new bone by performing essential functions in cellular processes that are vital for bone growth and healing. The purity, crystal structure, morphology, particle size, and surface characteristics of WH can be used for regenerative applications [25].

Bio-ceramics have been extensively employed for treating bone defects. Magnesium and hydrogen combined are important elements found in bone WH. WH is an essential part of biological hard tissues, such as the teeth and bones, in children. Synthesizing WH crystal is essential for simulating tissue composition and structure, making it easier to build bioinspired materials for drug delivery systems, scaffolds for tissue engineering, and medical implants. Magnesium WH is created when heterogeneous species are nucleated by the phospholipids in the bone [13]. Phosphatidylserine and phosphatidylinositol facilitate the mineralization of hydroxyapatite in bones. *In vitro*, the process explicates the role of WH and upregulates the genes involved in cell differentiation. Magnesium is one of the significant components that stimulate osteogenic and neurological properties [23]. This WH continually releases magnesium ions that influence mineralization, cell proliferation, and alkaline phosphatase (ALP) activity and are directly affected by magnesium ions. The higher concentration of calcium and magnesium ions results in a greater amount of protein adsorption due to the negative surface charge of WH, which is associated with the material's improved capacity for bone repair. It increases the synthesis of alkaline phosphate, which is essential for bone growth and mineralization [30]. Therefore, it possesses a significant value as a material for bone tissue engineering, drug delivery, and other biomedical applications.

Limitations of the study

WH receives less attention compared to hydroxyapatite, tricalcium phosphate, and other calcium phosphate minerals. Hence, the lack of comprehensive reports may hinder its properties and efficiency in biomedical applications. The synthesis of WH in the appropriate phase is not adequately explored due to the limited understanding of the material. This study provided an explanation of the structural, morphological, and hemostatic properties of WH. A thorough comprehension of biocompatibility characteristics, specifically in terms of blood and cellular compatibility, is necessary to evaluate the material's potential in physiological environments. Furthermore, in order to comprehensively examine the effects of the biological environment on medical advancements, it is imperative to conduct animal studies to analyze both the positive and negative aspects.

## Conclusions

An analysis was conducted on the impact of fabrication methods on the characteristics of WH. Through an XRD pattern, it was found that WH and calcium magnesium phosphate crystalline phases were present. The presence of phosphate peaks in Raman spectroscopy indicates the functional group properties of the materials. Small spherical morphologies were found through FE-SEM. The elemental composition of WH, such as calcium, magnesium, and phosphorus, was observed through EDS and elemental mapping techniques. The morphological analysis revealed a rapid hemostatic response. The hydrothermal synthesis of WH showed sharp crystalline indexing and distinct Raman spectra, which indicates enhanced WH features compared to coprecipitation methods for hemostatic and regenerative applications.

## References

[1] Elliott JC, Mackie PE, Young RA (1973). Monoclinic hydroxyapatite. Science.

[2] Li X, Zhang F, Zhao D (2015). Lab on upconversion nanoparticles: optical properties and applications engineering via designed nanostructure. Chem Soc Rev.

[3] Yang YS, Yuan YZ, Zhang YP, Guo HC, Xue JJ (2023). Cinnamyl chalcone based AIE fluorescent probes for sensitive detection of hydrazine and its application in living cells. J Fluoresc.

[4] Mohd Roslan MR, Mohd Kamal NL, Abdul Khalid MF (2021). The state of starch/hydroxyapatite composite scaffold in bone tissue engineering with consideration for dielectric measurement as an alternative characterization technique. Materials.

[5] Chae T, Yang H, Leung V, Ko F, Troczynski T (2013). Novel biomimetic hydroxyapatite/alginate nanocomposite fibrous scaffolds for bone tissue regeneration. J Mater Sci Mater Med.

[6] Singh SS, Roy A, Lee B, Banerjee I, Kumta PN (2016). Synthesis, characterization, and in-vitro cytocompatibility of amorphous β-tri-calcium magnesium phosphate ceramics. Mater Sci Eng C.

[7] Sikder P, Ren Y, Bhaduri SB (2020). Microwave processing of calcium phosphate and magnesium phosphate based orthopedic bioceramics: a state-of-the-art review. Acta Biomater.

[8] Ratnayake JT, Mucalo M, Dias GJ (2017). Substituted hydroxyapatites for bone regeneration: a review of current trends. J Biomed Mater Res B Appl Biomater.

[9] Motoshima H, Goldstein BJ, Igata M, Araki E (2006). AMPK and cell proliferation-AMPK as a therapeutic target for atherosclerosis and cancer. J Physiol.

[10] Taktak R, Elghazel A, Bouaziz J, Charfi S, Keskes H (2018). Tricalcium phosphate-fluorapatite as bone tissue engineering: evaluation of bioactivity and biocompatibility. Mater Sci Eng C.

[11] Toosi S, Behravan J (2020). Osteogenesis and bone remodeling: a focus on growth factors and bioactive peptides. Biofactors.

[12] Pacheco-Quito EM, Ruiz-Caro R, Veiga MD (2020). Carrageenan: drug delivery systems and other biomedical applications. Mar Drugs.

[13] Kakani AK, Veeramachaneni C, Majeti C, Tummala M, Khiyani L (2015). A review on perforation repair materials. J Clin Diagn Res.

[14] Green JJ, Elisseeff JH (2016). Mimicking biological functionality with polymers for biomedical applications. Nature.

[15] Jang HL, Jin K, Lee J, Kim Y, Nahm SH, Hong KS, Nam KT (2014). Revisiting whitlockite, the second most abundant biomineral in bone: nanocrystal synthesis in physiologically relevant conditions and biocompatibility evaluation. ACS Nano.

[16] Jang HL, Lee HK, Jin K, Ahn HY, Lee HE, Nam KT (2015). Phase transformation from hydroxyapatite to the secondary bone mineral, whitlockite. J Mater Chem B.

[17] Tas AC (2016). Transformation of brushite (CaHPO4·2H2O) to whitlockite (Ca9Mg(HPO4)(PO4)6) or other CaPs in physiologically relevant solutions. J Am Ceram Soc.

[18] Batool S, Liaqat U, Hussain Z, Sohail M (2020). Synthesis, characterization and process optimization of bone whitlockite. Nanomaterials.

[19] Zhai S, Wu X, Xue W (2015). Pressure-dependent raman spectra of β-Ca3(PO4)2 whitlockite. Phys Chem Miner.

[20] Kizalaite A, Klimavicius V, Balevicius V (2023). Dissolution-precipitation synthesis and thermal stability of magnesium whitlockite. Cryst Eng Comm.

[21] Kizalaite A, Grigoraviciute-Puroniene I, Asuigui DR (2021). Dissolution-precipitation synthesis and characterization of zinc whitlockite with variable metal content. ACS Biomater Sci Eng.

[22] Batool S, Liaqat U, Babar B, Hussain Z (2021). Bone whitlockite: synthesis, applications, and future prospects. J Korean Ceram Soc.

[23] Liao HT, Tsai MJ, Brahmayya M, Chen JP (2018). Bone regeneration using adipose-derived stem cells in injectable thermo-gelling hydrogel scaffold containing platelet-rich plasma and biphasic calcium phosphate. Int J Mol Sci.

[24] Jeong J, Kim JH, Shim JH, Hwang NS, Heo CY (2019). Bioactive calcium phosphate materials and applications in bone regeneration. Biomater Res.

[25] Henkel J, Woodruff MA, Epari DR (2013). Bone regeneration based on tissue engineering conceptions - a 21st century perspective. Bone Res.

[26] Vezenkova A, Locs J (2022). Sudoku of porous, injectable calcium phosphate cements - path to osteoinductivity. Bioact Mater.

[27] Guo Y, Su Y, Gu R, Zhang Z, Li G, Lian J, Ren L (2020). Enhanced corrosion resistance and biocompatibility of biodegradable magnesium alloy modified by calcium phosphate/collagen coating. Surf Coat Technol.

[28] Burezq HA, Davidson MK (2021). Ecological intensification for soil management: biochar - a natural solution for soil from agricultural residues. Sustainable Intensification for Agroecosystem Services and Management.

[29] Ma Z, Bao G, Li J (2021). Multifaceted design and emerging applications of tissue adhesives. Adv Mater.

[30] Chen X, Wang M, Chen F (2020). Correlations between macrophage polarization and osteoinduction of porous calcium phosphate ceramics. Acta Biomater.

