# Correction: Exploration of Whitlockite Nanostructures for Hemostatic Applications

**DOI:** 10.7759/cureus.c183

**Published:** 2024-06-11

**Authors:** Abhay Kumar Jain V, Saheb Ali, Ramadurai Murugan, Chitra S

**Affiliations:** 1 Pharmacology, Saveetha Medical College and Hospital, Saveetha Institute of Medical and Technical Sciences, Saveetha University, Chennai, IND; 2 Periodontics, Saveetha Dental College and Hospitals, Saveetha Institute of Medical and Technical Sciences, Saveetha University, Chennai, IND; 3 Center for Global Health Research, Saveetha Medical College and Hospital, Saveetha Institute of Medical and Technical Sciences, Saveetha University, Chennai, IND; 4 Prosthodontics, Saveetha Dental College and Hospital, Saveetha Institute of Medical and Technical Sciences, Saveetha University, Chennai, IND

This article has been corrected as follows:

Figure 6 was mistakenly included twice as both Figure 6 and Figure 7. Figure 7 has been replaced with the correct image. The legend for Figure 7 was updated to read: The elemental mapping data of whitlockite synthesized using the coprecipitation method: (A) a mixture of all the elements, (B) calcium, (C) phosphate, (D) magnesium, (E) oxygen, and (F) carbon.

